# Enhancement of Low pH Stress Tolerance in Anthocyanin-Enriched Transgenic Petunia Overexpressing *RsMYB1* Gene

**DOI:** 10.3389/fpls.2018.01124

**Published:** 2018-08-21

**Authors:** Aung H. Naing, Deuk B. Lee, Trinh N. Ai, Ki B. Lim, Chang K. Kim

**Affiliations:** Department of Horticultural Science, Kyungpook National University, Daegu, South Korea

**Keywords:** abiotic stress, antioxidants, plant growth, relative gene expression, MYB transcription factor

## Abstract

We investigated whether the presence of anthocyanins in plants could contribute to low pH stress tolerance using anthocyanin-enriched transgenic petunia lines (PM2, PM6, and PM8) expressing *RsMYB1* and wild-type (WT) plants. We examined several physiological and biochemical factors and the transcript levels of genes involved in abiotic stress tolerance. A reduction in plant growth, including plant height and fresh weight, was observed when plants (PM2, PM6, PM8, and WT) were exposed to low pH (pH 3.0) conditions compared to growth under normal (pH 5.8) conditions. A small reduction in the growth of PM6 was observed, followed by that in PM2, PM8, and WT, reflecting the anthocyanin levels in the plants (PM6 > PM2 and PM8 > WT). An analysis of physiological and biochemical factors also supports the degree of low pH tolerance in the plants (PM6 > PM2 and PM8 > WT). In addition, an enhanced expression of the genes [*superoxide dismutase* (*SOD), catalase* (*CAT), peroxidase* (*POX), osmotin*, and *vacuolar H^+^-ATPase (V-ATPase*)] was observed in the transgenic lines (PM2, PM6, and PM8). The resultant of the enhanced transcript levels of the genes could promote antioxidant activities, proline content, and pH homeostasis involved in the mechanisms underlying abiotic stress tolerance in plants. These results suggest that anthocyanin-enriched plants overexpressing *RsMYB1* enhances low pH stress tolerance by elevating the transcript levels of the relevant genes.

## Introduction

Owing to the improper application of chemical fertilizers [such as excess application of nitrogen (N) fertilizers] and cultural practices (continuous monoculture), soil acidification is rapidly increasing worldwide and becoming a major threat to agriculture (Wu et al., 2013; Yang et al., 2013). Acidic soils due to the presence of high H^+^ result in poor crop growth and yield due to the reduction in water and nutrient uptake [phosphorus (P), calcium (Ca), magnesium (Mg), and potassium (K)] (von Uexküll and Mutert, 1995; Kamaluddin and Zwiazek, 2004; Yang et al., 2011; Bian et al., 2013). Recently, Zhang C.P. et al. (2014) claimed that P uptake by *Juglans regia* seedlings is lower at pH 3.0. In addition, lower uptakes of K, Ca, and Mg by plants at pH 3.5 than at pH 5.5 have been reported previously (Malkanthi et al., 1995; Arduini et al., 1998). Induction of oxidative stress via enhanced production of reactive oxygen species (ROS) due to low pH has also been reported (Martins et al., 2013) and found to have higher lipid peroxidation in the pH 4.0-treated *P. algarbiensis* shoots. Zhang et al. (2015) and Shi et al. (2006) observed that the accumulation of hydrogen peroxide (H_2_O_2_) and severe lipid peroxidation caused by acidic soil (low pH) is due to the decreased activities of antioxidant enzymes such as superoxide dismutase (SOD) and catalase (CAT) in rice and cucumber. From many studies, it is obvious that a low pH limits the uptake of nutrients and water necessary for plant growth and causes the generation of excess ROS that damage plant cells and growth, resulting in reduced crop yields. Therefore, it is important to develop plants that can resist low pH-induced excess ROS so that crop productivity can be maintained.

Antioxidants scavenge ROS and reduce the oxidative stress in several plant species (Hirschi et al., 2000; Tseng et al., 2007). The high survival abilities of anthocyanin-enriched plants that correlate to high antioxidant contents have been studied under abiotic and biotic stress conditions (Agati et al., 2011; Dehghan et al., 2014). Moreover, improved abiotic stress tolerance linked to enhanced expression of vacuolar H^+^-ATPase (V-ATPase), increased anthocyanin accumulation, and increased ROS-scavenging abilities has been demonstrated (Fini et al., 2011; Cheng et al., 2013; Dong et al., 2013; He et al., 2014; Nakabayashi et al., 2014; Naing et al., 2017). To overcome abiotic stress conditions, it is therefore important to produce anthocyanin-enriched plants that can produce antioxidants. The studies conducted by Cheng et al. (2013) and Lim et al. (2016) indicated that overexpression of the IbMYB1 and *RsMYB1* transcription factor (TF) enhances anthocyanin and antioxidant activity in potato and Arabidopsis. However, they did not investigate the role of MYB-induced anthocyanin in low pH tolerance. In addition, the high transcript level of the genes related to antioxidants and proline has also been observed in anthocyanin-enriched plants, particularly under abiotic stress conditions, and can effectively scavenge excess ROS and improve abiotic stress tolerances (Kamaluddin and Zwiazek, 2004; Wu et al., 2013; Yang et al., 2013). Recently, Naing et al. (2017) reported that an increase in the tolerance to abiotic stress in tobacco transgenic lines is linked to the presence of high anthocyanin content in the lines. In our previous work, we developed independent transgenic lines (PM2, PM6, and PM8) expressing *RsMYB1* that contain different anthocyanin levels, and two of the three lines have been recently reported (Ai et al., 2017). However, an association between anthocyanin enrichment and abiotic stress tolerance, particularly low pH stress tolerance, was not investigated in previous works. Hence, it is of interest to address the gaps in previous experiments.

In this study, we used different transgenic petunia lines (PM2, PM6, and PM8) containing anthocyanin and expressing *RsMYB1*. We studied the involvement of anthocyanin in the mechanism related to tolerating low pH by examining several factors, such as plant height, fresh weight, uptake of nutrient, and the transcript levels of relevant genes involved in abiotic stress.

## Materials and Methods

In our previous work, we produced three transgenic petunia lines (PM2, PM6, and PM8) expressing *RsMYB1* that contain different anthocyanin levels in the whole plant; the regulation of anthocyanin in PM2 and PM6 has been recently reported (Ai et al., 2017). In this study, we used the lines (T_0_-PM2, T_0_-PM6, and T_0_-PM8) as plant materials to be examined for low pH stress tolerance.

### Production of T_2_ Generation

First, T_0_-PM2, T_0_-PM6, and T_0_-PM8 lines grown in a greenhouse were self-pollinated to obtain T_1_ seeds. Subsequently, the resultant seeds were grown in the greenhouse and the seedlings were screened based on the visual anthocyanin phenotype. Second, the screened T_1_ lines were further self-pollinated to obtain the final T_2_ seeds, and the resultant seeds were used as the plant material source for further experiments.

### Measurement of Anthocyanin Content and ROS-Scavenging Activity

Seeds of the T_2_ lines (PM2, PM6, and PM8) and wild-type (WT) plants were grown in the greenhouse for 8 weeks, and the 8-week-old T_2_ plants with an anthocyanin phenotype and WT plants were selected for the analysis of total anthocyanin content and ROS-scavenging activity.

To determine the anthocyanin content, fresh leaves (fifth top leaf of T_2_ lines and WT, ∼500 mg) were collected and ground to a fine powder. The powder was immediately transferred to an extraction solution and the extract was incubated at 4°C overnight and centrifuged at 13,000 rpm also at 4°C for 20 min. The supernatant containing anthocyanin was analyzed using a spectrophotometer (Shimadzu, Kyoto, Japan) as described in an earlier work (Ai et al., 2017).

To determine the ROS-scavenging activity, fresh leaves (fifth top leaf, 2.0 g) were collected from the T_2_ lines and WT plants, and the ROS-scavenging activity was measured using 2,2′-azino-*bis*(3-ethylbenzothiazoline-6-sulfonic acid) diammonium salt (ABTS) and 1,1-diphenyl-2-picrylhydrazyl (DPPH) assays (George et al., 2012; Lim et al., 2016). For each measurement, three biological samples were used for each of the T_2_ lines and WT plants. The analysis was repeated thrice.

### *In vitro* Seed Germination of T_2_ Lines (*PM2, PM6, and PM8*) and WT Plants

The T_2_ seeds were immersed in sterile distilled water containing 0.05% sodium hypochlorite solution (Yuhan Co., Ltd., Seoul, South Korea) and 0.01% Tween 20 (Duchefa, Haarlem, Netherlands) for about 5 min. The seeds were then rinsed with sterile distilled water at least three times and were air-dried in a sterilized filter paper until excess water was removed. The seeds were cultured in an MS basal medium containing 3% sucrose and 0.7% agar. The cultures were then incubated at 25 ± 2°C with a photoperiod of 16 h and a light intensity of 50 μmol m^-2^ s^-1^ for 45 days.

### Exposure of Transgenic Lines to Low pH Condition

Among the 45-day-old plants, the T_2_ transgenic lines (PM2, PM6, and PM8) showing the anthocyanin phenotype and a uniform size were selected. The WT plants were selected based on the size of the transgenic lines. For the low pH treatment, the selected plants were exposed to an acidic condition by culturing in the MS medium by adjusting the pH (to the low pH of 3.0) for 20 or 30 days, whereas the MS medium was adjusted to pH 5.8 and was used as the control. The culture conditions were the same as described above. Each treatment contained 60 plants (30 plants each for 20-day and 30-day measurements) and three replicates were used.

After the exposure of the plants to the low pH (3.0) and control conditions (pH 5.8) for 20 or 30 days, 20 plants from each of the T_2_ transgenic lines (PM2, PM6, and PM8) and WT plants were subjected to low pH (3.0) and control (pH 5.8) conditions for 20 or 30 days and were randomly selected for physiological, biochemical, and relevant gene expression analyses to determine various factors, including plant height, fresh weight, relative water content (RWC), total chlorophyll content, ROS-scavenging activities, uptake of nutrient, and antioxidant- and proline-related gene expression. The measurements were performed three times, and the data represent the means of three replicates.

### Measurement of RWC

Relative water content was measured using the 5^th^ leaf from the top of the transgenic lines (PM2, PM6, and PM8) and WT plants were subjected to low pH (3.0) and control (pH 5.8) conditions for 20 or 30 days. Fresh leaf weight was immediately recorded as soon as the leaves were excised from the plants. They were floated in deionized water at 4°C overnight, and their rehydrated weights were recorded again. Finally, leaves were oven-dried at 70°C overnight and dry leaf weight was recorded. The formula for measuring RWC is as follows: RWC = (fresh weight - dry weight)/(rehydrated weight - dry weight). Five leaves each from the transgenic lines (PM2 and PM6) and WT plants were used to measure the RWC and the analysis was repeated three times.

### Measurement of Chlorophyll Content

Following the stress, chlorophyll content was measured using the fifth leaf from the top of the transgenic lines (PM2, PM6, and PM8) and WT plants subjected to low pH (3.0) and control (pH 5.8) conditions for 20 or 30 days, following Baek et al.’s work (Baek et al., 2012). Briefly, the leaves were homogenized in 15 mL of 100% methanol, and the homogenate was filtered through two layers of cheesecloth. This was followed by centrifugation at 3,000 ×* g* for 10 min. The total chlorophyll content in the supernatant was measured and calculated using the formula described by Wellburn (1994).

### Assay of ROS-Scavenging Activity

Following the stress, the ROS-scavenging activities (using DPPH and ABTS assays) were measured using the fifth leaf from the top from the transgenic lines (PM2, PM6, and PM8) subjected to low pH (3.0) and control (pH 5.8) conditions for 20 or 30 days. The protocols were identical to those used in the above experiments (Kim et al., 2014; Lim et al., 2016). Three biological samples were used for each of the transgenic lines (PM2, PM6, and PM8) and WT plants. The analysis was repeated thrice.

### Uptake of Nutrients

Exactly 1 g of dried leaf tissue was collected from the transgenic lines (PM2, PM6, and PM8) and WT plants subjected to low pH (3.0) and control (pH 5.8) conditions for 30 days to analyze the uptake levels of the nutrients (N, Ca, K, Mg, P, and Al). The analysis was carried out as described by Cataldi et al. (2003). There were three samples per treatment and three replicates.

### RNA Extraction and Gene Expression Analysis by Quantitative Real Time-PCR (qRT-PCR)

The transcript levels of proline-related gene (*osmotin*), three antioxidant genes (*SOD, CAT*, and *POX*), and the *V-ATPase* gene expressed in the transgenic lines and WT plants subjected to the low pH (pH 3.0) and control conditions (pH 5.8) for 20 or 30 days were investigated. Total RNA was isolated from 100 mg of leaf tissue per sample using TRI Reagent^TM^ solution (Ambion, United States). One microgram of total RNA and an oligo dT20 primer were used for reverse transcription (ReverTra Ace-α-^®^ Toyobo, Japan). The transcript levels of the genes related to the reference gene (tubulin gene) were measured using a StepOnePlus Real-Time PCR system (Thermo Fisher Scientific, Waltham, MA, United States) (Naing et al., 2017). The primers and PCR conditions for the detected genes are listed in **Supplementary Tables 1, 2**. Three samples per line were used, and the analysis was repeated three times.

### Statistical Analysis

Data were collected until day 30 from the start date of the experiment, and statistical analysis was performed using SPSS version 11.09 (IBM Corporation, Armonk, NY, United States). The results are presented as means ± SE. The Duncan’s multiple range test was used to separate the means, and the significance was set at *P* < 0.05.

## Results

### Measurement of Anthocyanin Content and ROS-Scavenging Activity in T_2_ Generations

**FIGURE 1:**
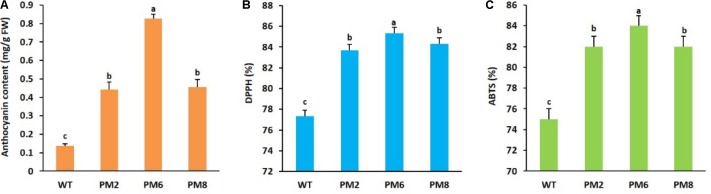
Comparison of **(A)** anthocyanin content and ROS-scavenging activities, **(B)** DPPH, and **(C)** ABTS, in PM2, PM6, PM8, and WT plants grown in the greenhouse for 8 weeks. Error bars indicate the standard error (SE) of the means.

The T_2_ transgenic plants (PM2, PM6, and PM8) obtained by successive self-pollination of T_0_ and T_1_ plants displayed anthocyanin phenotypes and their segregation ratios based on the anthocyanin phenotype followed the Mendelian law [3 (red):1 (green)] (data not shown). However, the total leaf anthocyanin content in PM6 was significantly higher than that in PM2 and PM8, whose contents were not significantly different from each other, and were followed by that of WT plants (**Figure 1A**). When investigating the ROS-scavenging activity by using ABTS and DPPH assays, as observed for anthocyanin contents, the activities were significantly higher in PM6 followed by PM2, PM8, and WT plants (**Figures 1B,C**), indicating the direct association between the ROS-scavenging activity and the anthocyanin content.

### Assessment of Plant Growth Parameters Under Low pH Acidic Condition

**FIGURE 2:**
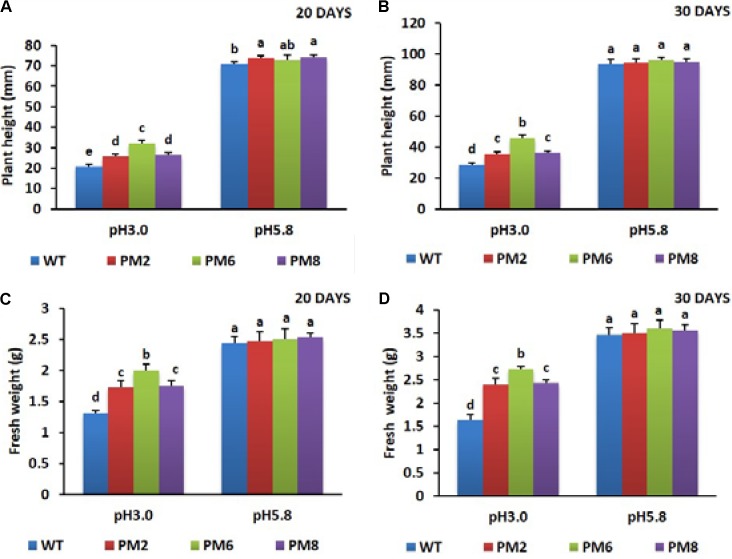
Comparisons of plant height **(A,B)** and fresh weight **(C,D)** of PM2, PM6, PM8, and WT plants under different pH conditions. Data were taken on days 20 **(A,C)** and 30 **(B,D)** after starting the experiments. Error bars show the standard error (SE) of the mean.

Growth responses of the T_2_ transgenic lines (PM2, PM6, and PM8) and WT plants subjected to acidic (pH 3.0) and normal (pH 5.8) growing conditions were evaluated 20 or 30 days after the treatments began. For the pH 5.8, no significant differences in plant height and fresh weight were observed in these plants until 30 days. However, when they were subjected to the low pH of 3.0 for 20 days, their shoots showed signs of water deficiency and significant inhibition of plant growth parameters, such as plant height and fresh weight (**Figures 2A,B**). When they were continuously allowed to grow for 30 days (**Figures 2C,D**), plant growth was gradually promoted but was still lower than that in the normal growing condition (pH 5.8). Among the plants, the PM6 plants were found to be more tolerant to low pH stress than PM2 and PM8, followed by the WT plants, because the growth parameters evaluated in the PM6 plants were significantly higher than that in PM2 and PM8, and those in WT plants were the lowest (**Figures 2A–D**). Overall, the low pH stress condition significantly inhibited the growth of the plants compared to the normal growing condition (pH 5.8). Furthermore, severe toxicity was clearly observed in the WT plants followed by the PM2, PM8, and PM6 plants. Therefore, the presence of anthocyanin content in the plants can be linked to the degree of acidity stress tolerance.

### Measurement of Nutrients Uptake in Plants After Acidic Stress Treatments

**FIGURE 3:**
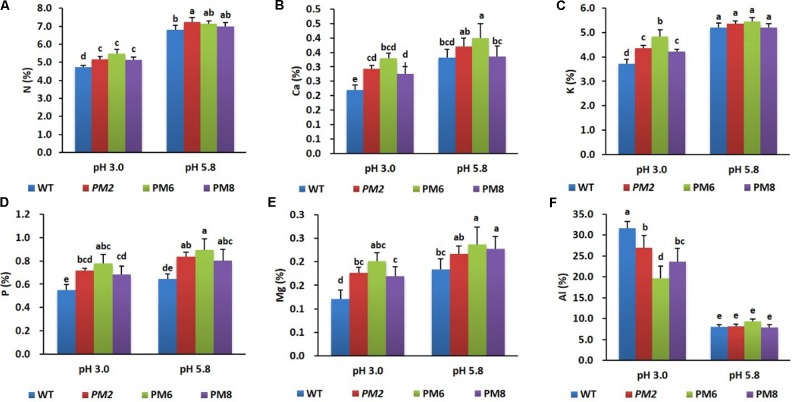
Comparison of nutrient uptake [**(A)**, N; **(B)**, Ca; **(C)**, K; **(D)**, P; **(E)**, Mg; and **(F)**, Al] by the plants (PM2, PM6, PM8, and WT) under different pH conditions. Data were taken on day 30 after starting the experiments. Error bars indicate the standard errors (SE) of average results.

After being exposed to a low pH condition (pH 3.0) for 30 days, uptake levels of the nutrients (N, Ca, K, Mg, P, and Al) by the plants (PM2, PM6, PM8, and WT) were measured relative to the normal growing condition (pH 5.8). Most of the nutrient uptake by the plants was significantly different under the two conditions. The percentage accumulation of the nutrients (except Al) was found to be the highest in PM6 followed by that in PM2, PM8, and WT plants (**Figures 3A–F**).

### Assessment of Chlorophyll Content and RWC

**FIGURE 4:**
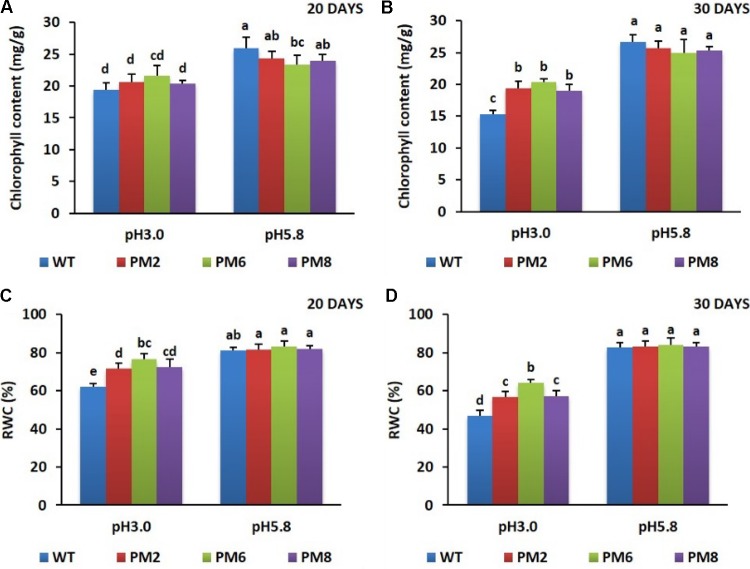
Comparison of chlorophyll content **(A,B)** and RWC **(C,D)** in PM2, PM6, PM8, and WT plants after they were exposed to different pH conditions for 20 and 30 days. Data were taken on days 20 **(A,C)** and 30 **(B,D)** after starting the experiments. Error bars show the standard error (SE) of the mean.

After being exposed to low pH (pH 3.0) for 20 days, reduction in the chlorophyll content and the RWC was observed in the plants compared to those grown at pH 5.8 (**Figures 4A,C**), and a significant reduction was observed when the treatment was extended up to 30 days (**Figures 4B,D**). The lowest amounts were detected in the WT plants followed by PM2 and PM8, and PM6 plants under the low pH stress conditions (**Figures 4A–C**).

### Assessment of ROS-Scavenging Activity

**FIGURE 5:**
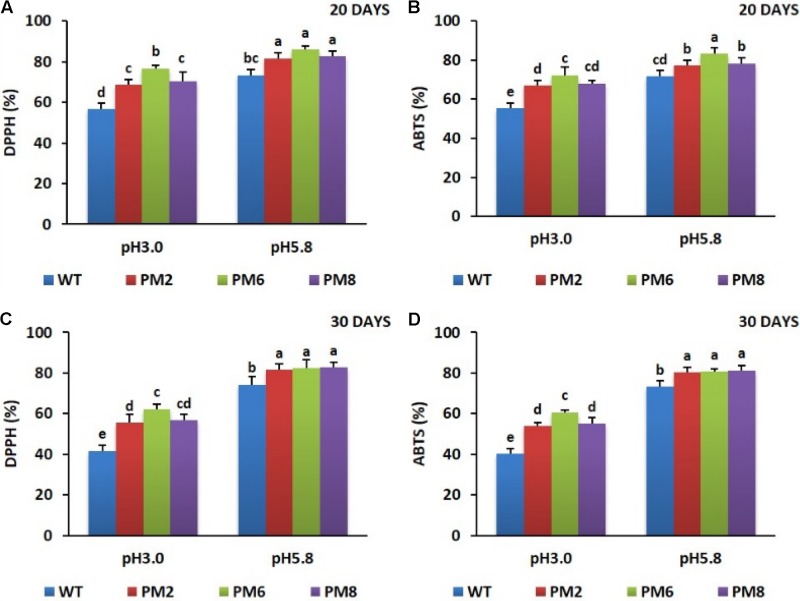
Comparison of ROS-scavenging activities, DPPH **(A,C)** and ABTS **(B,D)**, in PM2, PM6, PM8, and WT plants after they were exposed to different pH conditions for 20 days **(A,B)** and 30 days **(C,D)**. Data were taken on days 20 and 30 after starting the experiments. Error bars show the standard error (SE) of the mean.

Similar to what was observed for chlorophyll content and RWC, a significant reduction of ROS-scavenging activity (ABTS and DPPH) in the plants were noticed in the low pH condition as compared to the pH 5.8 condition for both 20 and 30 days. The levels of reduction in these plants were associated with the presence of anthocyanins because a more severe reduction was observed in the WT plants than what was seen in the PM2 and PM8 plants followed by the PM6 plants (**Figures 5A–D**).

### Expression Pattern of Relevant Genes

**FIGURE 6:**
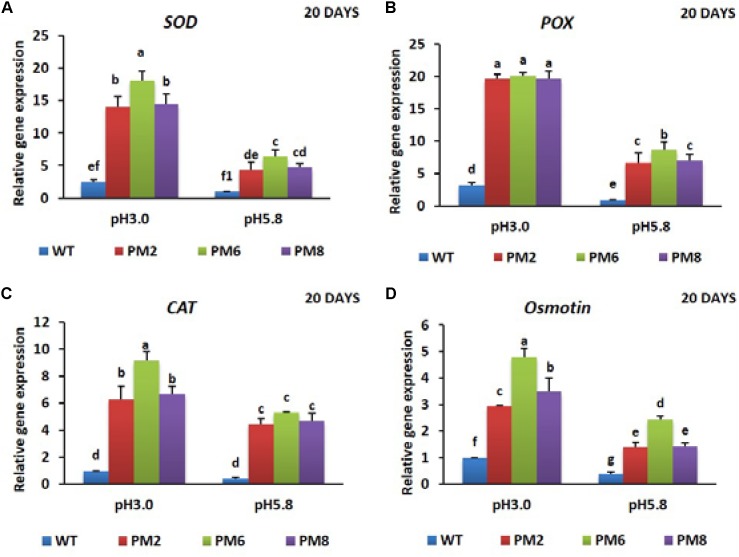
Expression analysis of antioxidant-related genes, *SOD* **(A)**, *POX* **(B)**, *CAT* **(C)**, and proline-related gene [*Osmotin*; **(D)**], in PM2, PM6, PM8, and WT plants after they were exposed to different pH conditions for 20 days. Data were taken on day 20 after starting the experiments. Error bars show the standard error (SE) of the mean.

**FIGURE 7:**
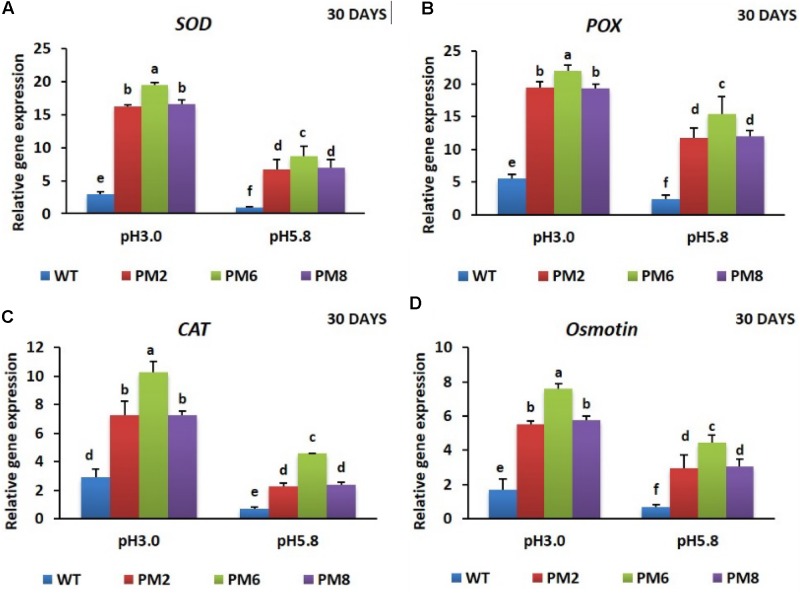
Expression analysis of antioxidant-related genes, *SOD* **(A)**, *POX* **(B)**, *CAT* **(C)**, and proline-related gene [*Osmotin*; **(D)**], in PM2, PM6, PM8, and WT plants after they were exposed to different pH conditions for 30 days. Data were taken on day 30 after starting the experiments. Error bars show the standard error (SE) of the mean.

**FIGURE 8:**
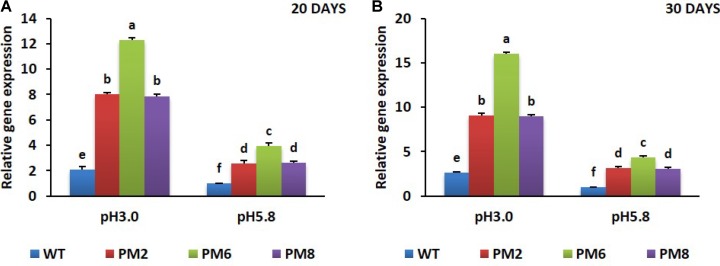
Expression analysis of *V-ATPase* gene in PM2, PM6, PM8, and WT plants after they were exposed to different pH conditions for 20 days **(A)** and 30 days **(B)**. Data were taken on days 20 and 30 after starting the experiments. Error bars show the standard error (SE) of the mean.

To reveal the molecular mechanisms underlying the degree of acidic stress tolerance in the plants (PM6 > PM2 and PM8 > WT), the expression levels of the antioxidant-related genes (*SOD, CAT*, and *POX*), proline-related gene (*osmotin*), and *V-ATPase* gene, which are involved in abiotic stress tolerance, were measured in the plants subjected to both pH conditions for 20 or 30 days using qRT-PCR. The transcript levels of the tested genes were significantly elevated at low pH (3.0) as compared to that at normal pH (5.8) for all plants at both 20- and 30-day periods, and expression levels in PM2 were the highest followed by that in PM6, PM8, and WT plants, indicating that the increases were in line with the level of tolerance to low pH (PM6 > PM2 and PM8 > WT) (**Figures 6A–D, 7A–D, 8A,B**).

## Discussion

In this study, low pH (3.0) inhibited plant growth in both the transgenic lines (PM2, PM6, and PM8) and WT plants relative to the normal growing condition (pH 5.8) at 20 or 30 days after treatments by displaying a reduction in the uptake of nutrients (N, P, K, Ca, and Mg) and water, and decreases in total chlorophyll contents and ROS-scavenging activity. The inhibition of plant growth by acidic soils (low pH soil) has been previously reported in several plant species (Arduini et al., 1998; Yang et al., 2011; Bian et al., 2013; Martins et al., 2013; Zhang C.P. et al., 2014; Long et al., 2017), and the results obtained in this study were in line with the results of the previous studies, all of which showed the inhibitory effects of low pH on plant growth via reduction of nutrient and water uptake by plants as well as reduced ROS-scavenging activities and chlorophyll content. In this study, uptake of Al by the plants was higher than that of other nutrients (N, P, K, Ca, and Mg) in plants grown at pH 3.0 than those grown at pH 5.8, supporting the results of previous findings that a low pH increased the solubility of Al and decreased the availability of P, Ca, and Mg (George et al., 2012; Kochian et al., 2015; Li et al., 2015). The reduction of total chlorophyll content, a main component of photosynthesis, would be due to the lower uptake of the major nutrients because photosynthesis in the leaves is subjected to acidity stress as acidic soils are limited by the nutrients (N, Ca, and Mg) (Ellsworth and Liu, 1994; Long et al., 2017). Moreover, an increase in Al content may also, in part, affect plant growth by indirectly inhibiting chlorophyll synthesis by competing with Mg, an integral part of the chlorophyll molecule needed for the binding sites in the plasma membrane of roots (Ali et al., 2011). In addition to the reduction of nutrients and water uptake and the lower chlorophyll content in the low pH-treated plants, the plants might be exposed to excess production of H_2_O_2_ and lipid peroxidation (Foyer and Shigeoka, 2011; Martins et al., 2013; Zhang C.P. et al., 2014; Long et al., 2017). This can cause oxidative damage to chlorophyll synthesis and thereby inhibit photosynthesis as well as nutrient and water uptake, thereby affecting plant growth. Reduced ROS-scavenging activity was also found in plants grown at pH 3.0 (relative to pH 5.8), reduction of the ROS-scavenging activity might be that the plants actively scavenged for excess ROS produced by pH 3.0; nevertheless, the reduction of plant growth was likely due to the presence of insufficient ROS-scavenging activity in the plants.

Despite no significant differences in plant growth and fresh weight between the transgenic plants (PM2, PM6, and PM8) and WT plants at pH 5.8, the reduction of plant growth and fresh weight was more serious in WT plants when grown at low pH (3.0), followed by PM2, PM8, and PM6, indicating slightly higher tolerance of PM6 to low pH followed by PM2, PM8, and WT (PM6 > PM2 and PM8 > WT). In fact, differences in their tolerances are associated with the levels of chlorophyll, nutrient and water uptake, and ROS-scavenging activity in the plants because these were consistently higher in PM6 than that seen in PM2, PM8, and WT plants.

The order of tolerance to low pH by the plants (PM6 > PM2 and PM8 > WT plants) and the presence of the higher nutrient uptake as well as chlorophyll content and ROS-scavenging activities (PM6 > PM2 and PM8 > WT plants) could be due to the presence of different anthocyanins in the plants (PM6 > PM2 and PM8 > WT plants); high anthocyanin accumulation provides greater ROS-scavenging ability to protect the plants from oxidative damages caused by the production of excess ROS during different abiotic stresses (Hossain et al., 2009; Cheng et al., 2013; Nakabayashi et al., 2014; Qi et al., 2015; Yuan et al., 2015), and might help uptake adequate amounts of nutrients and minimize the loss of chlorophyll and thereby plant growth. The presence of higher RWC in the order (PM6 > PM2 and PM8 > WT plants) could also be due to the presence of higher ROS-scavenging activity in the plants (PM6 > PM2 and PM8 > WT plants) (**Figure 5**) due to the ability to detoxify heavy metals such as Al in this study and enable the plants to take up water easily (Hirschi et al., 2000; Tseng et al., 2007). Persistence of the higher ROS-scavenging activity in the plants (PM6 > PM2 and PM8 > WT plants) till 30 days at low pH demonstrated that PM6 was the most tolerant to a low pH condition relative to PM2 and PM8, which can tolerate the condition slightly more than the WT plants.

The expression levels of the antioxidant (*SOD, CAT*, and *POX*) and proline genes (*osmotin*) responsible for scavenging or neutralizing ROS were determined in all plants subjected to both pH conditions to identify the mechanisms underlying the different tolerance levels of the plants to the low pH of 3.0 (PM6 > PM2 and PM8 > WT plants). The expression levels of these genes in the plants (PM6 > PM2 and PM8 > WT plants) are positively correlated to their degree of low pH tolerances (PM6 > PM2 and PM8 > WT plants), and the greater inhibition of WT growth compared with those of the PM2, PM6, and PM8 plants suggests that the induction of antioxidant and proline activity in the WT plants are lower than that seen in the PM2, PM6, and PM8 plants. Significant elevation of the genes in all plants under the low pH of 3.0 than under pH 5.8 might be due to the fact that the plants enhanced their expression levels to defend against ROS formation caused by the low pH. However, gene induction in the WT plants seem to be insufficient to properly scavenge ROS and the higher expression of the genes in the PM2, PM6, and PM8 plants could be explained by the presence of anthocyanin regulated by *RsMYB1*. Perhaps, *RsMYB1* may directly bind to the genes involved in antioxidant and proline production. High anthocyanin accumulation is linked to high antioxidant and proline activities (Winkel-Shirley, 2002; Agati et al., 2011; Dehghan et al., 2014; Naing et al., 2017). Therefore, this result supports the hypothesis that the higher tolerance to low pH conditions in the plants (PM6 > PM2 and PM8 > WT) depends on their expression levels of the genes related to proline and antioxidant production.

The involvement of V-ATPase in abiotic stress tolerance has been recognized in many plant species because its enhanced expression was observed under different abiotic stressors (Golldack and Deitz, 2000; Fukada et al., 2004; Baisakh et al., 2012; Zhang X.H. et al., 2014). Similar to these studies, in this study, the transcript levels of V-ATPase were found to be higher in low pH stress conditions than in normal growing conditions (pH 5.8), and that observed in PM6 was the highest followed by PM2, PM8, and WT. Transcriptional upregulation of V-ATPase in the plants was associated with low pH stress tolerance. This might occur because of the role of V-ATPase in regulating vacuolar pH (around pH 5.5) to prevent maximal lumen acidification in the vacuoles of the plant species (Dietz et al., 2001; Muller and Taiz, 2002). Another explanation for this is that its enhanced expression levels would promote the sequestration of excess toxic ions in vacuoles to aid in the adaptation to abiotic stress (Dietz et al., 2001). Dong et al. (2013) also reported that overexpression of V-ATPase from apples enhanced drought tolerance in transgenic tobacco. Similarly, overexpression of V-ATPase also significantly affected salt tolerance in *Arabidopsis thaliana* (He et al., 2014).

## Conclusion

This study characterized the involvement of anthocyanin in imparting tolerance to low pH stress using anthocyanin-enriched transgenic lines expressing *RsMYB1* and WT plants. The results suggest that the presence of higher anthocyanin enables tolerance to low pH stress by elevating the transcript levels of the genes that are involved in abiotic stress tolerance in plants. As *RsMYB1-*induced anthocyanins improve tolerance to low pH, its overexpression in horticultural and agricultural crops would improve both anthocyanin production and tolerance to abiotic stress.

## Author Contributions

AN and DL designed the study, conducted the experiment, and wrote the manuscript. KL and TA assisted in the conduction of the experiment. CK supervised experiments at all stages and performed critical revisions of the manuscript. All authors read and approved the final manuscript.

## Conflict of Interest Statement

The authors declare that the research was conducted in the absence of any commercial or financial relationships that could be construed as a potential conflict of interest.
