# Supplementary material for: Enhancement of Low pH Stress Tolerance in Anthocyanin-Enriched Transgenic Petunia Overexpressing RsMYB1 Gene
Source: Front Plant Sci. 2018 Aug 21;9:1124. doi: 10.3389/fpls.2018.01124 (PMC6111200; doi:10.3389/fpls.2018.01124)
Supplement: Supplementary file 1 [file Table_1.docx]

Supplementary file

Table 1. Primer sequences for qRT-PCR of antioxidant- and proline-related genes.

| Gene | Accession No. | | Primer sequences (5′-3′) |
| --- | --- | --- | --- |
| *SOD* | EU342358 | | F; GCCAGCTTTGAAGATGAACGA  R; GCCTAATGCTCTTCCCACCAT |
| *CAT* | U93244 | | F; GATGACAAGATGCTTCAAACTCGTA  R; CACTTTGGAGCATTAGCAGGAA |
| *POX* | D11396.1 | | F; ACTGCTCCGTCACCCAAAAC  R; GCCCTGGTTCCTGCTTAAGTC |
| *Osmotin* | X95308 | | F; ACTATCGAGGTCCGAAACAACTG  R; GCATTGATCACCCAAGTTTGG |
| *V-ATPase* | | AF220611.1 | F; GCTGATGCTCTTCGTGAGGT  R; CGATACGTCCAGCTCGTTCA |
| *Tubulin* | | SGN-U207876 | F; TGGAAACTCAACCTCCATCCA  R; TTTCGTCCATTCCTTCACCTG |

Table 2. PCR conditions for qRT-PCR of antioxidant- and proline-related genes.

| Genes | PCR conditions: |
| --- | --- |
| *SOD* | 95°C (10 min) → [95°C (30 s) → 59°C (30 s)] × 40 cycles → 95°C (15 s) → 59°C (30 s) |
| *CAT* | 95°C (10 min) → [95°C (30 s) → 59°C (30 s)] × 40 cycles → 95°C (15 s) → 59°C (30 s) |
| *POX* | 95°C (10 min) → [95°C (30 s) → 60°C (30 s)] × 40 cycles → 95°C (15 s) → 60°C (30 s) |
| *Osmotin* | 95°C (10 min) → [95°C (30 s) → 59°C (30 s)] × 40 cycles → 95°C (15 s) → 59°C (30 s) |
| *V-ATPase* | 95°C (10 min) → [95°C (30 s) → 59°C (30 s)] × 40 cycles → 95°C (15 s) → 59°C (30 s) |
| *Tubulin* | 95°C (2 min) → [95°C (20 s) → 60°C (40 s)] × 30 cycles → 72°C (1 min) → 59°C (5 m) |
